# Laparoscopic omentectomy in primary torsion of the greater omentum: report of a case

**DOI:** 10.1186/s40792-019-0618-5

**Published:** 2019-05-09

**Authors:** Jun Kataoka, Toshikatsu Nitta, Masato Ota, Yuko Takashima, Yuta Yokota, Kensuke Fujii, Takeshi Higashino, Takashi Ishibashi

**Affiliations:** 1 Department of Surgery, Gastroenterological Center, Shunjukai Shiroyama Hospital, 2-8-1 Habikino Habikino-city, Osaka, 583-0872 Japan; 2 Department of Internal Medicine, Gastroenterological Center, Shunjukai Shiroyama Hospital, Osaka, Japan; 3 Department of General and Gastroenterological Surgery, Osaka Medical College Hospital, Osaka, Japan

**Keywords:** Primary torsion of the omentum, Acute abdomen, Laparoscopy

## Abstract

**Background:**

Torsion of the greater omentum is a rare cause of acute abdominal pain in adults and children. It is very difficult to make a correct diagnosis of torsion clinically because it mimics other acute pathologies; however, the preoperative diagnosis can be easily confirmed with the use of computed tomography (CT). Herein, we report a case of laparoscopic omentectomy for primary torsion of the omentum, which was not improved by conservative treatment.

**Case presentation:**

A 50-year-old Japanese man presented to our hospital with acute right lower quadrant abdominal pain of a few hours’ duration. Routine blood tests showed a white blood cell count of 8900/mm^3^, and the C-reactive protein (CRP) level was 8.13 mg/dl. Contrast-enhanced CT scan of the abdomen revealed twisting of the omentum with a local mass of fat density and fluid distributed in a whirling oval-shaped mass pattern at the right flank and iliac fossa. Therefore, the patient was admitted to our hospital based on a diagnosis of omental torsion.

The patient was treated with conservative treatment with analgesics, anti-inflammatories, and antibiotics. Although his symptoms were ameliorated, his laboratory and radiological findings worsened. We performed laparoscopic omentectomy 6 days after admission.

The resected omentum was 24 cm × 22 cm in size and was twisted and dark red in color, suggesting infarction. Histological analysis revealed that the specimen was ischemic and hemorrhagic omentum, accompanied by inflammatory infiltration.

The patient’s postoperative course was uneventful, and he was discharged 9 days later.

**Conclusion:**

This is a rare case of primary torsion of the greater omentum that was treated successfully with laparoscopic omentectomy. Considering the increase in surgical difficulty due to inflammation from prolonged torsion and the limited efficacy of conservative treatment, we conclude that surgical intervention is warranted as early as possible when torsion of the greater omentum is suspected.

## Background

Torsion of the greater omentum is a rare and unusual cause of acute abdominal pain in adults and children. Eitel et al. first described primary torsion of the omentum with no underlying pathology in 1899 [1]. It is very difficult to make a correct diagnosis of torsion clinically, because the disease symptoms mimic many other acute presentations. Although this rare disease is often not identified preoperatively, it may be accurately diagnosed with the use of computed tomography (CT) [2–4].

Currently, the first-line treatment of torsion of the greater omentum is conservative pharmacotherapy (analgesics, anti-inflammatories, and antibiotics) and observation, with the surgical treatment initiated immediately if the patient’s clinical, laboratory, and radiological findings worsen.

Herein, we report a case of laparoscopic omentectomy in primary torsion of the omentum which was not improved by conservative treatment.

## Case presentation

**Fig. 1:**
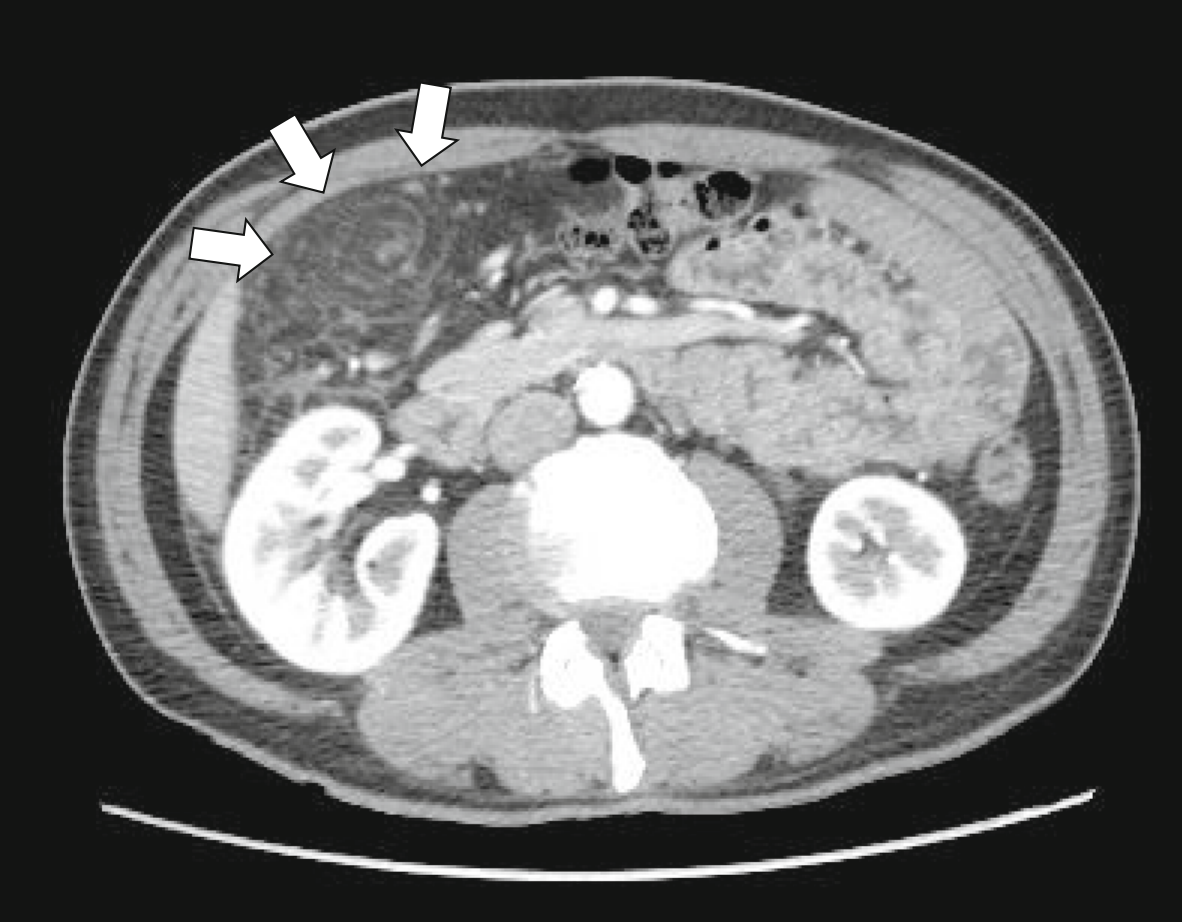
Preoperative abdominal computed tomography scan. Twisting of the omentum with a local mass of fat density and fluid in a whirling oval-shaped mass pattern at the right flank and iliac fossa (white arrow) was observed. The fatty mass is situated between the transverse colon and the gallbladder and contains hyperattenuating streaks

A 50-year-old Japanese man presented to the Department of Gastroenterological Center, Shunjukai Shiroyama Hospital with acute right lower quadrant abdominal pain of a few hours’ duration. Physical examination revealed that he was 172 cm tall, weighed 65 kg, and had a body mass index (BMI) of 21.8 kg/m^2^. The abdominal pain was localized without rebound tenderness or guarding while bowel sounds were normal. The patient had no associated nausea, vomiting, or diarrhea. His medical history was unremarkable with no previous abdominal operations or problems. Routine blood tests showed that his white blood cell count was 8900/mm^3^ and the C-reactive protein (CRP) level was 8.13 mg/dl (Table 1). Contrast-enhanced CT scan of the abdomen revealed twisting of the omentum with a local mass of fat density and fluid distributed in a whirling oval-shaped mass pattern at the right flank and iliac fossa (Fig. 1). The fatty mass was situated between the transverse colon and the gallbladder and contained hyperattenuating streaks.

**Table 1:** Laboratory findings

Peripheral blood		Blood chemistry		Serological tests	
WBC	8900/μL	TP	6.8 g/dL	CRP	**8.13** mg/dL
RBC	473 × 10^4^/μL	Alb	4.2 g/dL	HBsAg	(−)
Hb	15.0 × 10^4^/μL	T-Bil	1.0 mg/dL	HBsAb	(−)
Hct	42.9%	AST	15 U/L	HCVAb	(−)
Plt	22.8 g/dL	ALT	15 U/L	Coagulation	
		ALP	197 U/L	PT	12.0 s
		γ-GTP	38 U/L	PT-INR	1.05
		LDH	146 U/L	APTT	27.6 s
		BUN	12.2 mg/dL		
		Cre	0.78 mg/dL		
		Na	138 mEq/L		
		K	4.7 mEq/L		
		Cl	101 mEq/L		
		CPK	80 U/L		

Routine blood test showed that his white blood cell count was 8,900/mm^3^ and the C-reactive protein(CRP) level was 8.13mg/dl

Therefore, the patient was admitted to our hospital, based on a diagnosis of the omental torsion. At first, he was treated with conservative treatment because his vital signs were stable and his symptoms were non-specific and mild. Although his symptoms were ameliorated by oral analgesics, anti-inflammatory drugs, and prophylactic antibiotics, laboratory tests 5 days after admission showed a decreased leukocytosis (white blood cell count 5500/mm^3^) but increased CRP (18.49 mg/dl). A second CT scan showed that the twisting of the omentum with a local mass of fat density and fluid had been retained; moreover, the fat density and fluid were worsened. Therefore, laparoscopic omentectomy was performed 6 days after admission.

**Fig. 2:**
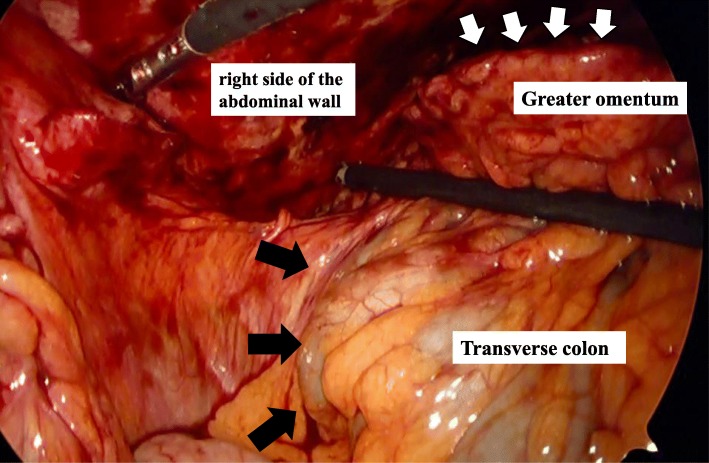
Surgical findings. During abdominal exploration, a solid ischemic and hemorrhagic portion of the right greater omentum (white arrow) was found adhered to the right side of the abdominal wall and transverse colon (black arrow)

**Fig. 3:**
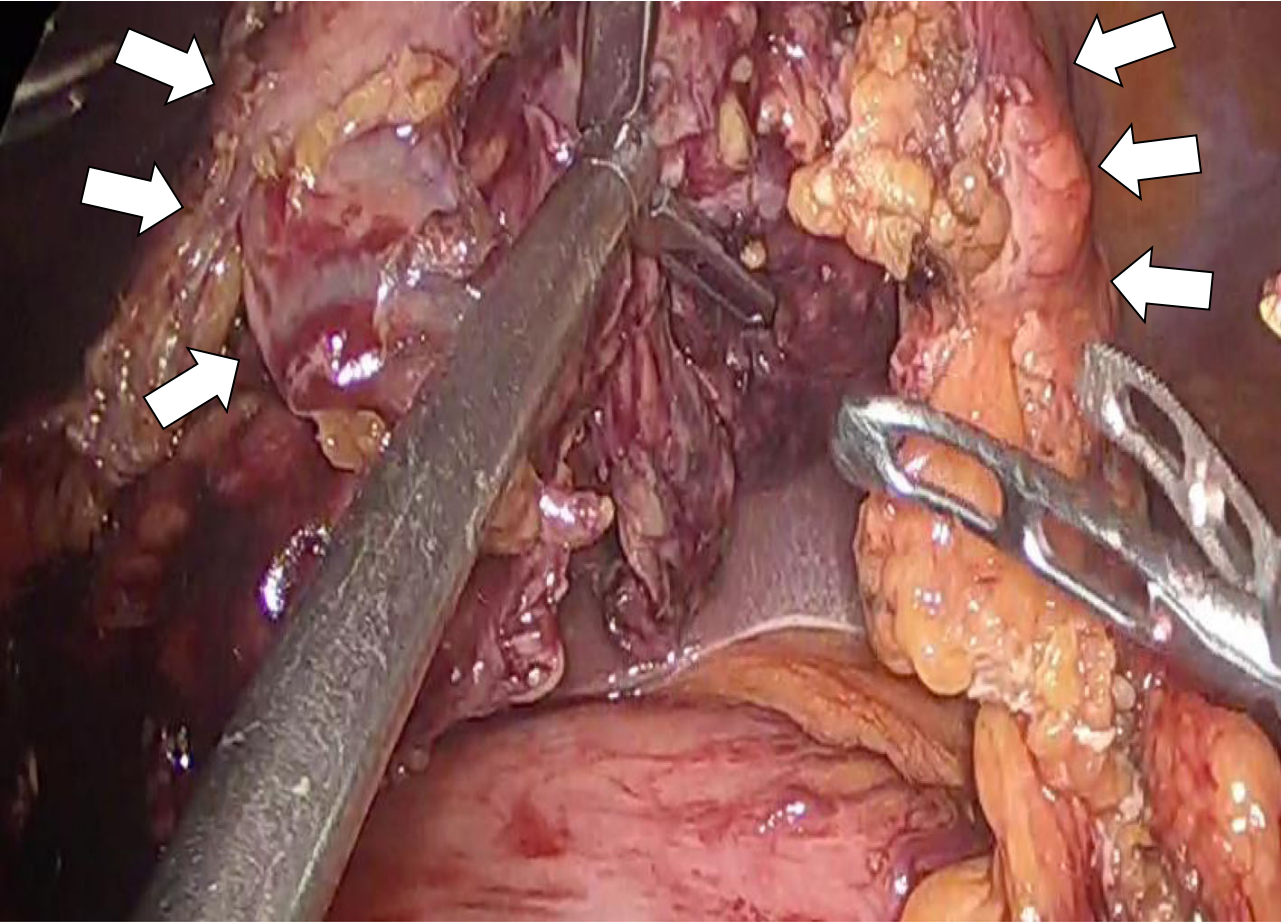
Surgical findings. The white arrow shows the free torsion after entering the omental bursa and resecting the adhesion between the transverse colon and omentum

The patient was placed in the supine position under general anesthesia. Initially, the abdominal cavity was reached using a 12-mm trocar from the left of the umbilicus. Four accessory trocars (5 mm each) were placed in the right upper quadrant, left upper quadrant, left lower quadrant, and above the pubis symphysis. During abdominal exploration, a solid ischemic and hemorrhagic portion of the right greater omentum was found adhered to the right side of the abdominal wall and transverse colon (Fig. 2). Although this portion was adhered to the transverse colon, the appendix and gallbladder were normal, and Meckel diverticulum was not present. Then we entered the omental bursa, and the adhesion between the transverse colon and omentum was resected. Therefore, this portion was completely free of adhesions due to the resection (Fig. 3). The specimen was retrieved in a bag through a small abdominal incision 5.0 cm in length above the umbilical trocar. The total operating time was 200 min, and the intraoperative blood loss was 100 ml.

**Fig. 4:**
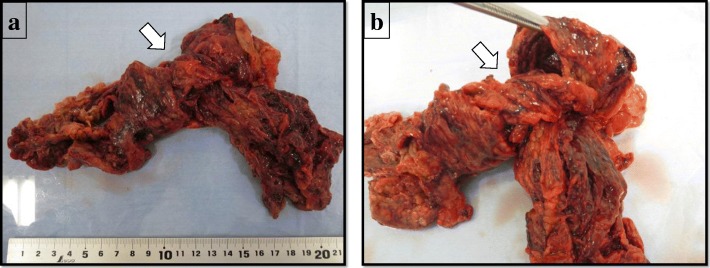
**a**, **b** Macroscopic findings. The 24 cm × 22 cm-sized specimen is twisted and dark red in color, suggesting infarction

Macroscopically, this specimen was 24 cm × 22 cm in size and was twisted and dark red in color, suggesting infarction (Fig. 4a, b). Histological analysis proved the specimen to be ischemic and hemorrhagic omentum, accompanied by inflammatory infiltration, confirming the diagnosis of omental infarction due to the torsion (Fig. 4a, b).

The patient’s postoperative course was uneventful, and he was discharged 9 days later.

## Discussion

Primary torsion of the greater omentum with no underlying pathology was first described by Eitel in 1898 [1], and segmentary infarct of the greater omentum was described for the first time by Bush in 1896 [5]. Less than 350 cases have been reported since the description by Bush, of which only 26 were treated using laparoscopic omentectomy. Torsion of the greater omentum accounts for only 1.1% of all cases of acute abdominal pain in adults and children [6, 7]. Omental torsion is primarily seen in the 30–50-year age group, with male predominance [8]. However, a few cases have been reported in children, and the estimated incidence of torsion of the greater omentum in children undergoing surgery for presumed appendicitis ranges between 0.024 and 0.1% [9, 10]. Torsion of the greater omentum is classified as primary or secondary, the latter being far more common [11]. Secondary torsion of the greater omentum is associated with a causative condition, such as adhesions, cysts, inguinal hernia, inflammation, previous laparotomy, trauma, or tumor [12, 13].

Secondary torsion is usually bipolar because torsion of the central portion occurs between two fixed points, resulting in formation of a narrow neck or pedicle somewhere in the continuity [14]. The etiology of primary torsion of the greater omentum is unclear, though a variety of factors have been proposed. Some anatomical malformations and anomalies are recognized as predisposing factors to torsion: presence in the greater omentum of tongue-like projections and bifid and accessory omentum, anomalous vascular blood supply, other vascular anomalies that modify the weight of the omentum, vascular kinking, or irregular omental pad, seen mostly in obese patients [2, 15, 16]. Obesity is a common risk factor leading to primary torsion, with one study documenting that almost 70% of patients with omental infarction were obese [17]. Precipitating factors include sudden movements, violent exercise, hyperperistalsis, trauma, and acute changes in body position [2]. In our case, although we cannot deny the possibility that the torsion was secondary to the adhesions observed at the transverse colon and the right side of the abdominal wall, we believe that these adhesions were mainly physiological because they were relatively easily released by exfoliation. Therefore, these adhesions were unlikely to be associated with the torsion of the omentum.

Patients present with acute onset of severe abdominal pain and tenderness that is localized to the right side of the abdomen in 80% of cases [18, 19]. Gastrointestinal symptoms, such as nausea, anorexia, and vomiting, are uncommon. Torsion of the greater omentum is very difficult to correctly diagnose preoperatively and clinically because it mimics other acute pathologies including acute appendicitis, acute cholecystitis, diverticulitis, perforated duodenal ulcer, abdominal wall hematoma, intestinal obstruction, and gynecologic diseases, as documented in nearly all cases reported in surgical literature [2]. An accurate preoperative diagnosis is reported in only 0.6–4.8% of cases [20]. When compared with appendicitis, this pathology has an incidence rate of 0.0016–0.37%, a ratio of less than 4 cases per 1000 cases of acute appendicitis [11, 21].

Therefore, the diagnosis of torsion of the greater omentum is usually made intraoperatively: however, the preoperative diagnosis may be accurately determined with the use of CT [3]. Reported CT scan findings in torsion of the greater omentum include a well-circumscribed, oval, or cake-like fatty mass with heterogeneous attenuation containing strands of soft tissue attenuation probably corresponding to fibrous bands or dilated thrombosed veins [17, 22]. Ultrasound analysis is also very sensitive for the preoperative diagnosis of torsion of the greater omentum in the absence of other abdominal signs. However, laboratory findings are not specific and, apart from slight leukocytosis, mimic other pathological abdominal conditions. Reliance on laboratory findings will likely delay diagnosis and contribute to an increasing degree and duration of the torsion of the greater omentum.

The treatment of patients with primary torsion of the greater omentum is controversial. Some reports have demonstrated that most patients successfully recover with conservative treatment because primary torsion of the greater omentum is a benign and self-limiting disease. Moreover, retraction, fibrosis, and complete resolution of the inflammatory process usually occur within 2 weeks [23]. However, surgical treatment is chosen when diagnosis is uncertain, or when the patient’s clinical, laboratory, and radiological findings worsen with conservative treatment including oral analgesics, anti-inflammatory drugs, and prophylactic antibiotic. In terms of diagnostic and definitive therapy, laparoscopy is the appropriate method [24, 25].

Furthermore, the advantages of laparoscopic techniques include the following: (1) complete examination of the abdominal cavity to confirm diagnosis, (2) facilitation of aspiration and washing of the peritoneum, and (3) minimization of surgical invasiveness, postoperative pain, and wound-related complications [26–29]. In our case, we chose surgical treatment because the conservative treatment was not effective; however, the surgery was made more difficult due to chronic and persistent inflammation, which may have worsened during conservative therapy.

## Conclusion

This is a rare case of primary torsion of the greater omentum that was treated successfully with laparoscopic omentectomy. Most patients successfully recover with conservative treatment; however, due to the possibility of increased surgical difficulty and the limitations of conservative treatment, we conclude that surgical treatment should be initiated as early as possible, rather than waiting for the outcome of conservative therapy when the diagnosis of torsion of the greater omentum is suspected.

## Abbreviations

CRP: C-reactive protein
CT: Computed tomography
